# Emerging bone marrow failure syndromes- new pieces to an unsolved puzzle

**DOI:** 10.3389/fonc.2023.1128533

**Published:** 2023-04-06

**Authors:** Simone Feurstein

**Affiliations:** Department of Internal Medicine, Section of Hematology, Oncology & Rheumatology, University Hospital Heidelberg, Heidelberg, Germany

**Keywords:** bone marrow failure, ADH5, ALDH2, DNAJC21, ERCC6L2, MECOM, early onset myeloid malignancies

## Abstract

Inherited bone marrow failure (BMF) syndromes are genetically diverse — more than 100 genes have been associated with those syndromes and the list is rapidly expanding. Risk assessment and genetic counseling of patients with recently discovered BMF syndromes is inherently difficult as disease mechanisms, penetrance, genotype-phenotype associations, phenotypic heterogeneity, risk of hematologic malignancies and clonal markers of disease progression are unknown or unclear. This review aims to shed light on recently described BMF syndromes with sparse concise data and with an emphasis on those associated with germline variants in *ADH5/ALDH2*, *DNAJC21*, *ERCC6L2* and *MECOM*. This will provide important data that may help to individualize and improve care for these patients.

## Introduction

Bone marrow failure (BMF) syndromes are defined by decreased production of one or more hematopoietic lineages, which leads to diminished or absent hematopoietic precursors in the bone marrow and subsequent cytopenia in the peripheral blood. BMF can be distinguished into an acquired form and an inherited form. The acquired form, which is likely caused by an autoimmune reaction (1), may be successfully treated with immunosuppressant regimens. Inherited BMF syndromes include a broad spectrum of heterogenous diseases such as Fanconi anemia, telomere biology disorders, Shwachman-Diamond syndrome, Diamond-Blackfan anemia, congenital cytopenia, immunodeficiency and others (2). In excess of 100 genes have been associated with inherited BMF to date (3–8). The first inherited BMF syndrome, Fanconi anemia, was described in 1927 by the Swiss pediatrician Guido Fanconi, who reported a family with three boys with physical birth defects and a condition resembling pernicious anemia (9). The first causative gene, *FANCC*, was successfully cloned in 1992 (10). A number of genes have emerged as new *bona fide* genes associated with the development of BMF in the past ten years: In 2014, variants in *ERCC6L2* were shown to cause autosomal recessive BMF and predisposition to myeloid malignancies (11). *MECOM* as causative gene for inherited BMF has been described in 2015 (12), but its association with a predisposition to hematologic malignancies was only reported three years later (13, 14). Homozygous/compound heterozygous variants in *DNAJC21* were linked to a Shwachman-Diamond-like BMF with additional telomeropathy-like features in 2016 (15). In 2020, a digenic ADH5/ALD2H2 deficiency causing severe BMF, early-onset myelodysplastic syndrome (MDS), short stature and intellectual disability was connected to the inability to detoxify formaldehyde (16).

Particularly for the recently described syndromes, data on disease mechanism, penetrance, overall risk of developing hematologic malignancies, and molecular or cytogenetic factors indicating a risk of worsening cytopenia, development of bone marrow dysplasia or leukemogenesis is sparse. This is an important and incomplete pillar for counseling patients and providing them with the most complete and up-to-date information specific to their underlying condition (17). Penetrance, risk of hematologic malignancy and phenotypic heterogeneity may influence the decision towards early (preventive) hematopoietic stem cell transplantation (HSCT) versus ‘watch and wait’ and a more specific follow-up program tailored to the early detection of clonal evolution and disease progression. Amino acid hotspots, genotype-phenotype correlations and disease mechanisms based on reported variants are crucial to determine the strength and validity of the underlying genetic diagnosis and the expected/predicted phenotype and course of disease. This review is therefore based on the recently described syndromes with germline variants in *ERCC6L2, MECOM, DNAJC21*, and *ADH5/ALDH2* that lack concise reviews at this point in time.

## ERCC6L2 acts as crucial non-homologous end joining factor

In 2014, whole-exome sequencing (WES) of three children and young adults (ages nine to nineteen years old) with BMF and neurological abnormalities (microcephaly, developmental delay) and a history of consanguinity revealed homozygous *ERCC6L2* variants in two index patients (11). *ERCC6L2* belongs to the Snf2-like *ERCC6* family, which also includes *ERCC6* and *ERCC6L.* Functional studies revealed that the molecular mechanism of ERCC6L2 deficiency is an impaired nucleotide excision repair mechanism and an increased amount of reactive oxygen species *via* a defect in the mitochondrial function of ERCC6L2 (11). The short ERCC6L2 isoform contains an N-terminal TUDOR and a C-terminal DEAD/DEAH ATP-helicase domain. Zhang et al. (18) later reported an alternative *ERCC6L2* transcript translating a new protein, Hebo (helicase mutated in BMF), which differs from the ERCC6L2 protein by an 850-amino acid sequence and an additional HEBO domain. Hebo is ubiquitously expressed and is recruited to sites of DNA damage (18). A subsequent study by Tummala et al. postulated the underlying mechanism as primary transcription deficiency rather than a DNA repair defect based on patients being defective in the repair of transcription-associated DNA damage leading to genomic instability (19). Liu et al. described that ERCC6L2 clusters with core subunit non-homologous end joining (NHEJ) genes. ERCC6L2-deficient cells were depleted upon treatment with γ-irradiation, zeocin and etoposide inducing double-strand breaks, lending itself to a similar, but less severe phenotype than that observed in cells lacking the NHEJ ligase LIG4. They could also demonstrate that *Ercc6l2*−/− mice were viable and ERCC6L2 deficiency resulted in an approximately 50% reduction in orientation-specific class switch recombination of antibody genes (20). A CRISPR-Cas9 screen against genotoxic agents also identified ERCC6L2 as a canonical NHEJ pathway factor (21). SFPQ, a member of the SFPQ-NONO complex that has recently been attributed a putative function in NHEJ, has been described as novel interaction partner of ERCC6L2 (22). Somatic *ERCC6L2* variants have been described in a variety of hematologic and solid malignancies, most commonly in patients with uterine corpus endometrial carcinoma. Upon treatment with radiotherapy, these patients showed a strikingly longer disease-free and overall survival than patients with wild-type ERCC6L2, indicating that ERCC6L2 loss may be clinically relevant (22). The most recent study described an impaired clonogenic capacity and erythroid differentiation in *ERCC6L2*-silenced HSPCs and a probable impact on mesenchymal stromal cells and their differentiation potential (23).

**Figure 1:**
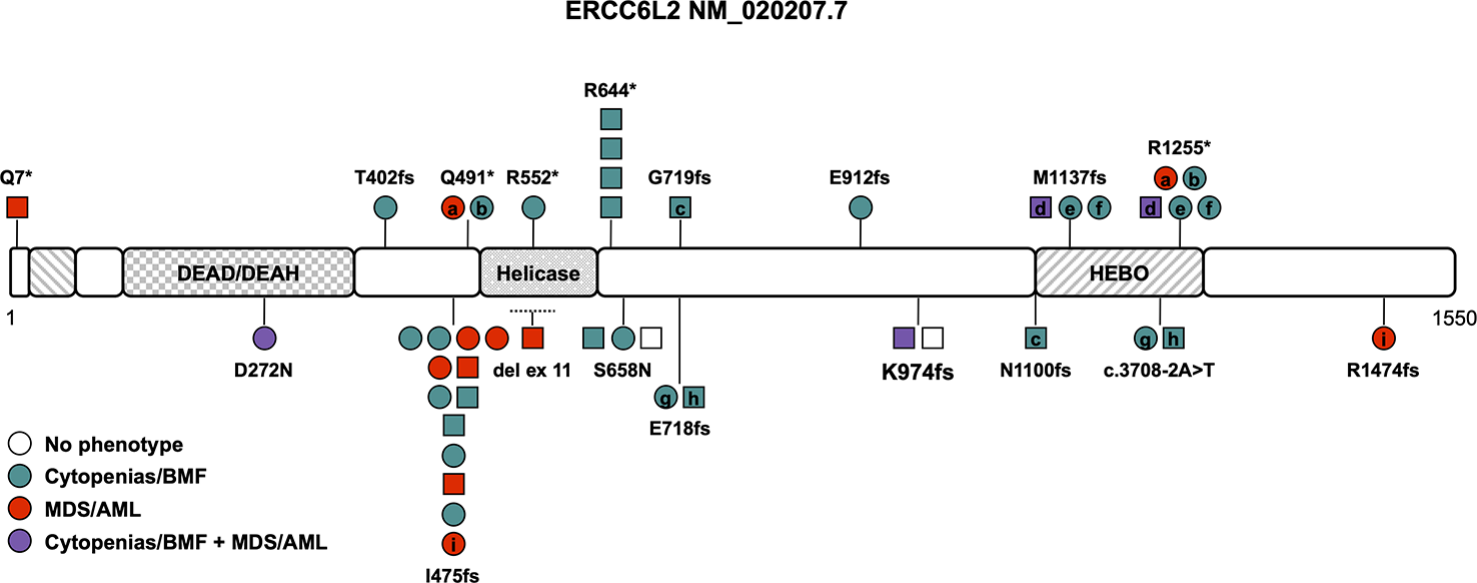
Schematic of the *ERCC6L2* transcript (NM_020207.7) and its protein domains with the location of all reported germline variants. Circles represent females and squares represent males. Symbols on the same horizontal level are individuals from the same family. Letters within the symbols indicate individuals with compound heterozygous genotype, while symbols without letters indicate homozygosity for the variant. The color fill depicts different phenotypes, no color fill designates healthy individuals carrying homozygous/compound heterozygous causative variants. The dotted line shows the location of the sole described copy number variant. AML, acute myeloid leukemia; BMF, bone marrow failure; HEBO, helicase mutated in bone marrow failure; MDS, myelodysplastic syndrome.

Consanguinity has been described in at least 8 of the 24 families (33%) reported to date (**Table 1**). The disease is caused by loss-of-function (LOF) variants and all but two variants, D272N and S658N (NM_020207.7) (7, 19), are truncating variants affecting both isoforms or just the long isoform with its HEBO domain (**Tables 1**, **S1** and **Figure 1**). Two variants, R644* and I475fs, have been found in more than one family and are present in the heterozygous state in gnomAD (https://gnomad.broadinstitute.org) with significant allele frequencies in the European (Non-Finnish) and Finnish subpopulation, respectively (**Table 1** and **Figure 1**). One copy number variant (CNV), a homozygous intragenic deletion of exon 11, has been reported as the causative allele in a patient with BMF (**Figure 1**) (24). The association with neurological abnormalities such as microcephaly, congenital mirror movements and developmental delay of various degrees was discovered in three studies (18, 24, 25) and may be part of the phenotype or could be an independent effect of the underlying consanguinity in these cases. Of note, ataxia, microcephaly, and developmental delay have also been described in diseases associated with variants in other NHEJ factors such as *ATM*, *MRE11*, *NBS1*, *NHEJ1*, *PRKDC*, *RAD50*, and *XRCC4* (26).

**Table 1:** Overview of emerging bone marrow failure syndromes including genetic and phenotypic features.

Gene	OMIM	inheritance/consanguinity/*de novo* occurence	mechanism of disease	amino acid hotspots	penetrance	phenotype	heterogeneity	genotype-phenotype association	age of onset	acquired somatic variants/disease progression	risk of heme malignancy, type	HSCT	PMID
***ADH5/ALDH2*** (NM_000671.4)	619151	digenic, AR for *ADH5*	LOF (missense and truncating SNVs), either heterozygous or homozygous for ALDH2*2	W322* described in all but one family; A278P described in seven out of 13 families	complete	cytopenia/BMF and/or MDS/AML [100%] short stature [100%] intellectual disability [100%] microcephaly [67%] abnormal skin pigmentation [58%] *(retinal degeneration, facial dysmorphia, skeletal, and endocrine abnormalities)*	high penetrance, severe BMF and MDS/AML often requiring HSCT	individuals with homozygous ALDH2*2 indicative of more severe (neurological) phenotype	MDS/AML: age 7 years (range 0 to 18, n=9)	73% with gain of the long arm of chromosome 1, other recurrent cytogenetic alterations included monosomy 7, trisomy 8 and 21p alterations	80%, MDS/AML	75%, after MDS/AML diagnosis	33147438 33355142 34458631
***DNAJC21*** (NM_001012339.3)	617052	AR, consanguinity in 57%	LOF (missense and truncating SNVs and CNVs)	K34E described in seven individuals from four families; R173* described in two families	complete	cytopenia/BMF and/or AML [100%] growth delay and/or short stature [95%] develomental delay, intellectual disability and/or neurological abnormalities [68%] skeletal abnormalities [63%] skin abnormalities [63%] microcephaly [42%] facial dysmorphia [37%] dental abnormalities [32%] osteopenia/osteoporosis [32%] (high) myopia, astigmatism and other visual field defects [32%] retinal (rod-cone) dystrophy and other retinal abnormalities [32%] pancreas lipomatosis/exocrine pancreatic dysfunction [26%]	although penetrance is high, spontaneous, intermittent or prolonged improvement of cytopenia has been reported	unknown	cytopenia/BMF: age 2 years (range 0 to 15, n=19) AML: ages 12 and 15 years (n=2)	complex karyotype, deletions of 17p13 and 20q and a derivative chromosome 15 with translocation t(1;15) described in four patients without hematologic malignancy	11%, AML^	21%, mostly due to severe BMF	35298850 27346687 35464845 30755392 29146883 28062395 29700810
***ERCC6L2*** (NM_020207.7)	615715	AR, consanguinity in 33%	LOF (truncating SNVs and CNVs), two missense variants (D272N and S658N)	R644* and F486fs (Finnish founder) described in more than one family	high (94%), two asymptomatic homozygotes	cytopenia, BMF [66%] MDS, AML (particularly acute erythroid leukemia) [31%] *(neurological abnormalities such as microcephaly, congenital mirror movements and intellectual disability)*	may vary from subtle intermittent cytopenia to severe BMF and/or early onset MDS/AML	unknown	cytopenia/BMF: age 14 years (range 2 to 47, n=24) MDS/AML: age 35 years (range 2 to 59, n=12)	complex or monosomal karyotype with loss of chromosomes 5, 7 or 17 or isolated monosomy 7, *TP53* variants (often multi-hit)	33%, MDS/AML	28%, either due to severe BMF or after the onset of MDS/AML	29633571 24507776 30936069 28815563 29987015 33209984 29146883 35969835 33510405 36156210
***MECOM*** (NM_004991.4)	616738	AD, *de novo* in 16%	LOF (missense and truncating SNVs and CNVs)	R938W, P948A and variants affecting the splice sites between exons 7 and 8 have been described in more than one family	high (96%) for any related features (RUS/other skeletal abnormalities, deafness, cytopenias/BMF)	cytopenia, BMF [80%] RUS [54%] brachy-, campto-, and clinodactyly and/or other finger abnormalities [38%] sensorineural hearing impairment/congenital deafness [20%] cardiac abnormalities [18%] prematurity, hydrops fetalis or polyhydramnios [13%] micro- or macrocephaly, structural brain abnormalities or intellectual disability/cognitive impairment [11%] patellar hypoplasia [10%] metatasus adductus, hallux valgus and other toe abnormalities [9%] clubfoot [7%] renal abnormalities [7%] MDS or MDS/MPN-U [5%] (*hip dysplasia, cleft palate, early-onset ischemic insults, facial dysmorphia, precocious puberty/gynecomastia in infancy, paralysis of the larynx/laryngomalacia)*	high penetrance, spontaneous, intermittent or prolonged improvement of cytopenias has been reported	distinct genotype-phenotype association- all but one variant associated with the co-presentation of RUS and hematologic disease cluster in the region spanning zinc fingers 8 and 9 (specific variants R969C/H/L, I971T, and Q965E have solely been associated with RUS without any other features)	cytopenia/BMF: at birth/in infancy (range 0 to 42, n=43) MDS or MDS/MPN-U: ages 37, 42 and 73 years (n=3)	unclear, translocation t(1;14)(q44;q32) described in one patient with MDS/MPN-U	5%, MDS/MPN^	50%, due to severe BMF	35020829 30536840 32064714 29200407 29519864 29439187 36082647 29097497 29540340 26581901 29146883 22972950 26554871 29496554 35484980 35150448

AD, autosomal dominant, AML- acute myeloid leukemia; AR, autosomal recessive; BMF, bone marrow failure; CNV, copy number variant; HSCT, hematopoietic stem cell transplantation; LOF, loss-of-function; MDS, myelodysplastic syndrome; MPN, myeloproliferative neoplasm; RUS, radioulnar synostosis; SNV, single-nucleotide variant; MPN-U, myeloproliferative neoplasm- unclassified.

Phenotypic features in parentheses and Italics are not clearly associated with the underlying BMF syndrome (yet).

^Only few patients reported.

There is no known genotype-phenotype association and the phenotype ranges from mild cytopenia to severe BMF in childhood and/or development of MDS/acute myeloid leukemia (AML) (**Table 1**). The overall penetrance is high with an estimate of 94% with two asymptomatic homozygotes still being very young (**Table 1**). Cytopenia and/or overt BMF develop early at an average age of 14 years and were reported in 24 out of 36 patients (66%, range 2 to 47 years (n=24), **Table 1**) (7, 11, 18, 19, 23, 25, 27, 28). The development of hematologic malignancies (MDS/AML) has been described in approximately 31% of *ERCC6L2* germline-mutated patients at an average age of 35 years (range 2-59 years (n=12), **Table 1**) (7, 19, 24, 28–30). Importantly, only one in four showed signs of cytopenia or BMF beforehand, which is in line with reports that cytopenia can be subtle, intermittent and go unnoticed. Close to all patients with MDS fell into high-risk groups with a complex karyotype or isolated monosomy 7 and co-occurring (often multi-hit) *TP53* alterations (**Table 1**) (7, 19, 24, 28–30). Several patients with acute erythroid leukemia have been reported, either progressing from MDS or as isolated disease, leading to the assumption that this AML subtype seems to be much more prevalent in *ERCC6L2* germline-mutated patients (**Table 1**) (28). Acute erythroid leukemia, defined by excess of maturation-arrested primitive erythroblasts, is a rare subtype of AML, occurring in about 3% of all AML patients (31). It is characterized by a significantly higher frequency of *TP53* variants (36%), especially bi-allelic/multi-hit *TP53* alterations with relatively lower somatic mutational burden compared to other AML subtypes (31, 32). While acute erythroid leukemia by itself does not seem to carry an additional prognostic impact as independent risk factor (33), its frequent association with complex karyotypes and multi-hit *TP53* alterations does confer to a dismal outcome in at least the subset of cases with these abnormalities (34). Cytogenetic abnormalities in the twelve *ERCC6L2* patients with AML and MDS presented often as a complex or monosomal karyotype with loss of chromosomes 5, 7 or 17 or isolated monosomy 7. In addition, multi-hit *TP53* alterations were reported in seven out of twelve patients with MDS/AML. An assessment of the allelic state of these *TP53* alterations was not performed (**Table 1**) (7, 19, 24, 28–30). The prognosis of MDS/AML in patients with *ERCC6L2* germline variants is poor, especially when progression to acute erythroid leukemia is noted, with no known survivors of this AML subtype so far (28). HSCT was performed in at least ten individuals (28%), either because of severe, transfusion-dependent BMF or after MDS/AML development (**Table 1**). Given the high frequency of monosomy 7, complex/monosomal karyotypes and *TP53* variants, which may be associated with disease progression and development of MDS/AML, HSCT should be considered early, especially when these aberrations are discovered in the context of clonal evolution and bone marrow dysplasia.

## MECOM deficiency serves as an example of a genotype-phenotype association

Heterozygous variants in *HOXA11* are known to cause radioulnar synostosis (RUS) (a congenital proximal fusion of the radius and ulna) with amegakaryocytic thrombocytopenia (RUSAT) (35). However, families with RUSAT without *HOXA11* variants were reported (36), suggesting that additional candidate genes/loci exist. Consequently, Niihori et al. (12) performed WES on three individuals with RUSAT without an identified variant in *HOXA11* and detected heterozygous missense variants in *MECOM* in all three patients. This was the first time RUSAT has been linked to variants in *MECOM*.

**Figure 2:**
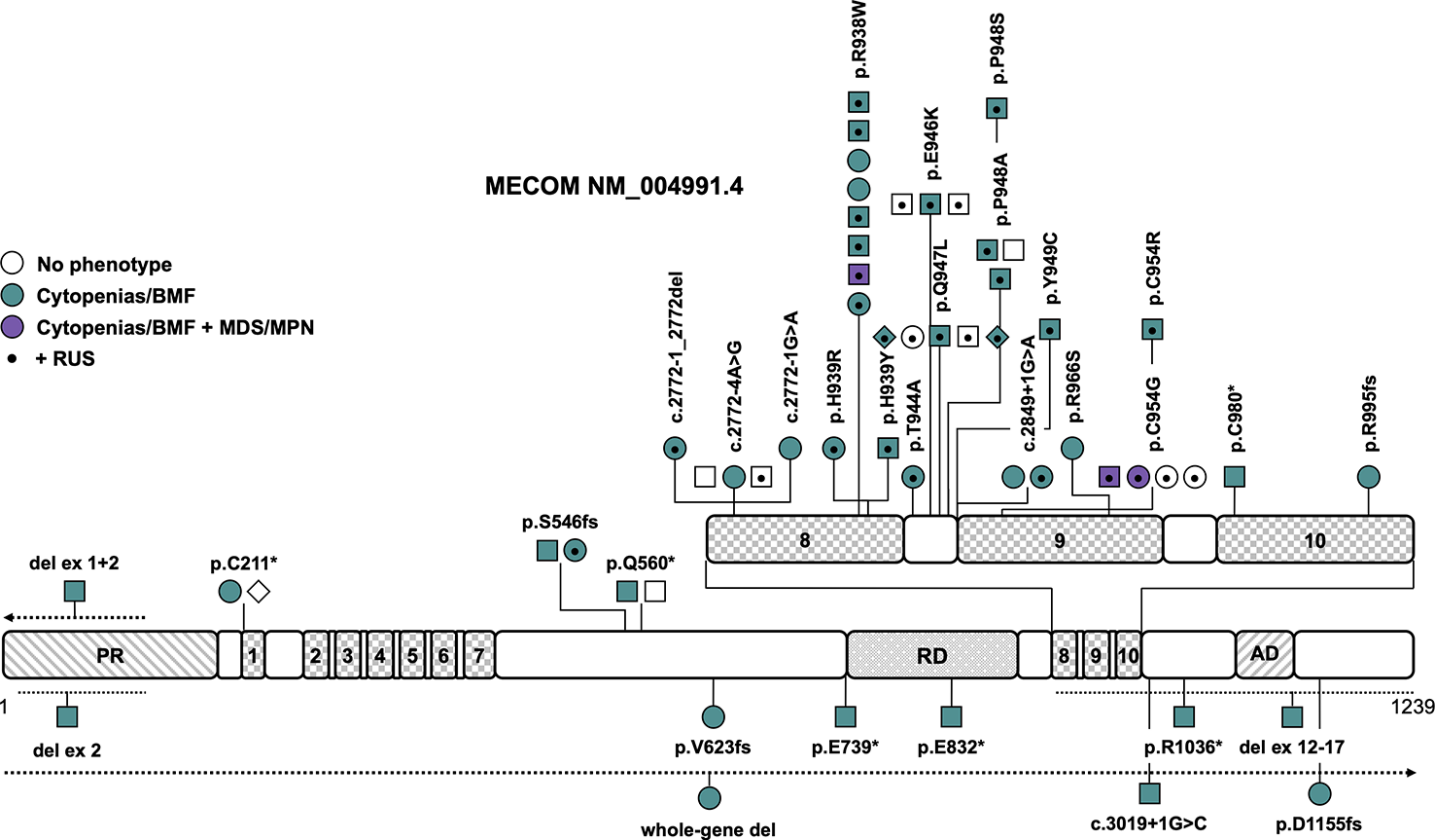
Schematic of the *MECOM* transcript (NM_004991.4) and its protein domains with the location of all reported germline variants. Checkered domains with the numbers 1 to 10 stand for zinc fingers 1 to 10. Circles represent females, squares represent males and diamonds represent unknown gender. Symbols on the same horizontal level are individuals from the same family. The color fill depicts different phenotypes, no color fill indicates healthy individuals carrying a causative variant. A dot in the middle of the symbol designates the presence of RUS in these individuals. The dotted lines show the location of the described copy number variants, the arrow denotes that the copy number variant extends in this direction. AD, acidic domain; BMF, bone marrow failure; MDS, myelodysplastic syndrome; MPN, myeloproliferative neoplasm; PR, PRDF1-RIZ domain; RD, repression domain; RUS, radioulnar synostosis.

The MDS1-EVI1 complex locus (MECOM) gives rise to several transcripts through alternative splicing of the N-terminus that encode at least three different isoforms: full length EVI1-145 kDa, EVI1-Δ324, which lacks zinc fingers 6 and 7, and MDS1-EVI1. MDS1-EVI1 comprises an N-terminal so-called PRDF1-RIZ homology domain, two C2H2 zinc finger DNA binding domains, one at the N-terminus including seven zinc fingers, and the other at the C-terminus including three zinc fingers, a proline-rich repressor domain and a small aspartate/glutamate-rich acidic region located in the C-terminal region (**Figure 2**) (37). MECOM acts as crucial transcription factor in hematopoiesis, playing an important role in the formation and self-renewal of long-term hematopoietic stem and progenitor cells (HSPCs) (38) as well as myeloid differentiation through interaction with transcription factors including GATA1 (39), SPI1 (40), RUNX1 (41) and others (42). The inversion or translocation of chromosome 3 drives inv(3)/t(3;3) AML *via* structural rearrangement of an enhancer that upregulates transcription of *EVI1*. It is associated with poor overall survival in AML patients and HSCT is usually mandated whenever possible (43). Furthermore, overexpression of *EVI1* has been reported in 6 to 11% of AML patients without 3q aberrations (44).

Later reports broadened the phenotype caused by germline *MECOM* variants, including BMF without RUS (7, 13, 45–50), predisposition to myeloid malignancies (14, 51), abnormalities of other organ systems (7, 13, 14, 45–47, 49–55), and RUS without any other phenotypic features (56). Causative variants include LOF variants that are scattered across the entire gene and missense variants that are solely located in zinc fingers 8 and 9 (**Figure 2** and **Tables 1**, **S1**). Confirmed *de novo* variants have been reported in 16% of patients (7, 48–51). The variants R938W and P948A (NM_004991.4) as well as variants affecting the splice sites between exons 7 and 8 have been described in more than one family (**Figure 2** and **Table 1**). Interestingly, there seems to be a rather distinct genotype-phenotype association — all but one variant associated with the co-presentation of RUS and hematologic disease cluster in the region spanning zinc fingers 8 and 9, which includes mostly missense, but also canonical and non-canonical splice variants (**Figure 2** and **Table 1**). Specific missense variants in zinc fingers 8 and 9 (namely R969C/H/L, I971T, and Q965E) have been described in 21 individuals from 6 families with RUS and finger malformations without hematological abnormalities (56). Little is known about this association of RUS with missense variants in zinc fingers 8 and 9. It was shown that the *Evi1* expression pattern is temporally and spatially restricted in mouse embryos with a transient expression in the emerging limb buds (57). *Junbo* mice with an *Evi1* variant affecting zinc finger 9 had extra digits on their forelimbs, suggesting that the C-terminal zinc finger domain may be relevant in digit development (58). Both the *MECOM* missense variants H939R and R969C have displayed attenuated suppression of TGFB1 (12, 56), which has been previously shown to play a role in digit formation during mouse development (59). LOF variants in *MECOM* seem to cause BMF but not RUS. CNVs have been described in four patients and were all confirmed or presumed *de novo* (**Figure 2**) (46, 48–50). One 751kb 3q26 microdeletion encompassing the entire *MECOM* gene and the pseudogene *EGFEM1P* was reported in one patient with BMF without other phenotypic features (48). Intragenic deletions of exons 1 + 2 (49), exon 2 (50) and exons 12-17 (46), affecting zinc finger and acidic domains, were reported in three patients with BMF at birth and a range of congenital skeletal and/or heart abnormalities. The intragenic deletion of exons 1 and 2 extended to other genes and also included the telomere biology gene *TERC*, so that specific phenotypic features cannot be attributed to either gene in this case (49). Overall penetrance of any related features (RUS/other skeletal abnormalities, deafness, cytopenia/BMF) is high at an estimated 96% (**Table 1**). Cytopenia/BMF was present in 80% of patients with an average age of onset at birth/in infancy (n=43) (7, 12–14, 45–55, 60). Although cytopenia can be severe and present at birth, even leading to intrauterine death, and may require early HSCT, three cases (5%) with spontaneous resolution or improvement of cytopenia/BMF have been reported (13, 45, 51). RUS was the most frequent non-hematopoietic feature in 54% of patients (7, 12–14, 51–55, 60), followed by brachy-, campto-, and clinodactyly and/or other finger abnormalities in 38% of patients (7, 12, 13, 45, 46, 51–53). Other frequent abnormalities included sensorineural hearing impairment/congenital deafness in 20% (12–14, 51), cardiac abnormalities such as atrial/ventricular septal defects, tetralogy of Fallot, aortic coarctation, pulmonary stenoses/atresias, pulmonary venous return anomaly, patent ductus arteriosus and myocardial atrophy in 18% (7, 13, 47, 49–51, 54) and prematurity, hydrops fetalis or polyhydramnios in 13% of patients (12, 47, 50, 53–55). Less frequent phenotypic features are micro- or macrocephaly, structural brain abnormalities or developmental delay/cognitive impairment in 11% (12, 13, 46, 51, 52), patellar hypoplasia (13, 14), metatarsus adductus, hallux valgus and other toe abnormalities (13, 14) in 9% and clubfoot (7, 51, 52) and renal abnormalities (7, 13, 55) in 7% of patients each (**Table 1**). Other features such as hip dysplasia (13, 52, 53), cleft palate (12, 13, 49), early-onset ischemic insults (13, 14, 51), facial dysmorphia (7, 49, 51), precocious puberty/gynecomastia in infancy (13), and paralysis of the larynx/laryngomalacia (45, 49) have been described in only two to three individuals and consequently the association with germline *MECOM* variants may not be entirely clear or proven in these cases. Three patients (5%) were reported to develop hematologic malignancies, specifically MDS with refractory cytopenia with multilineage dysplasia at 37 years (51), MDS with excess blasts-2 at 73 years with interstitial deletion of the long arm of chromosome 9 (14) and MDS/myeloproliferative disease- unclassifiable at 42 years with a translocation t(1;14)(q44;q32) (**Table 1**) (14). All patients reportedly had a history of thrombocytopenia or BMF with earlier onset (14, 51). HSCT has been performed in 27 out of 55 patients (50%) because of severe BMF.

## DNAJC21 deficiency- a new Shwachman-Diamond syndrome-like disorder with telomeropathy aspects

**Figure 3:**
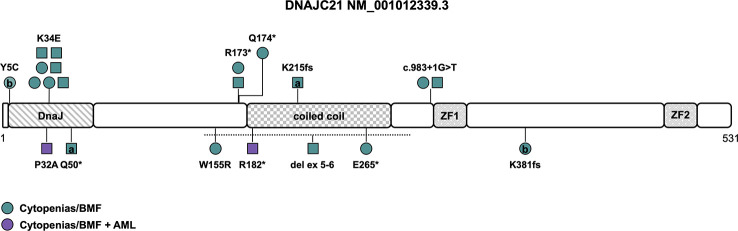
Schematic of the *DNAJC21* transcript (NM_001012339.3) and its protein domains with the location of all reported germline variants. Circles represent females and squares represent males. Symbols on the same horizontal level are individuals from the same family. Letters within the symbols indicate individuals with compound heterozygous genotype, while symbols without letters indicate homozygosity for the variant. The color fill depicts different phenotypes. The dotted line shows the location of the sole described copy number variant. AML, acute myeloid leukemia; BMF, bone marrow failure; ZF, zinc finger.

*DNAJC21* is ubiquitously expressed and encodes a protein with 531 amino acids, containing a highly conserved N-terminal DnaJ molecular chaperone homology domain, a central coiled coil region as well as two zinc fingers (**Figure 3**). The first studies in yeast showed that it functions together with the cytoplasmic zinc finger protein Znf622 to stimulate the ATPase activity of the Hsp70 chaperone protein Hspa8, thereby initiating the removal/recycling process of Arx1, a ribosome maturation factor (61, 62). In 2016, Tummala et al. screened a cohort of 28 unrelated individuals with BMF and syndromal features by WES and identified 3 individuals with homozygous variants in *DNAJC21*. By targeted re-sequencing of *DNAJC21* in patients with similar phenotype, a fourth patient with a homozygous *DNAJC21* variant was found (15). Functional studies on patient-derived lymphoblastoid cell lines implicated involvement of DNAJC21 in rRNA biogenesis and 60S ribosome maturation — thereby resembling the function of SBDS — leading to decreased interaction with HSPA8, ZNF622 and PA2G4 and increased cell death in patients with DNAJC21 deficiency (15). So far, 19 patients from 14 different families have been described in the literature (7, 15, 63–67), in 8 families (57%) a history of consanguinity was reported (**Table 1**). There is no confirmed case of a *de novo* variant in *DNAJC21* reported to date.

Causative variants include missense variants, particularly within the N-terminal DnaJ-domain affecting the amino acids 5, 32 and 34 (Y5C, K34E, P32A, NM_001012339.3), with two of those located in the universally conserved HPD motif (H33-P34-D35), which is essential for stimulation of ATPase activity. The K34E variant is the most common variant described in seven individuals from four families (**Figure 3** and **Tables 1**, **S1**) (64, 65), reversing the surface charge of a key amino acid adjacent to the HPD motif and also likely disrupting the interaction with HSPA8 (68). The P32A variant potentially alters the fold of the HPD motif, disrupting the interaction with HSPA8 and stimulation of its ATPase activity (68). Truncating variants encompassing nonsense, frameshift and canonical splice site variants (**Table S1**) are predicted to undergo nonsense mediated decay, leading to significant reduction of DNAJC21 protein expression (7, 15, 65–67). While most patients were homozygous for a causative *DNAJC21* variant, two individuals were found to be compound heterozygous (7, 66). Besides single-nucleotide variants, one individual with a homozygous intragenic deletion of exons 5 and 6 was reported as well (**Figure 3** and **Table 1**) (65).

While some features such as exocrine pancreatic dysfunction are consistent with a (classic) Shwachman-Diamond phenotype (as has been described in patients with bi-allelic inactivation of *SBDS* and less likely of *EFL1* or heterozygous variants in *SRP45* (69)), other features such as skin hypopigmentation, dental and retinal abnormalities seem to resemble characteristics of telomeropathies (64). There is no known genotype-phenotype association. The penetrance of a hematologic phenotype in the sense of single- or multiple lineage cytopenia/BMF seems to be complete (**Table 1**). The average age of onset for BMF is two years (range 0 to 15 years, n=19). Spontaneous, intermittent or prolonged improvement of cytopenia was reported in at least four patients (64–66), while four patients (21%) needed a HSCT (**Table 1**). AML developed in two patients (11%) at the age of twelve and fifteen years, respectively (15, 67). Somatic cytogenetic or molecular alterations were unknown or not reported. However, one case of acute megakaryoblastic leukemia was described (15). Other cytogenetic abnormalities, including a complex karyotype (7), a derivative chromosome 15 with translocation t(1;15) (65), a deletion of 17p13 (64) and a deletion of 20q (64) have been reported in patients with BMF without hematologic malignancy and consequently their significance in disease progression and development of MDS/AML is unclear. Growth delay and/or short stature as the most frequent non-hematopoietic feature has been described in all but one patient (95%). Other frequent abnormalities included developmental delay/intellectual disability and/or neurological abnormalities (68%), skeletal abnormalities (particularly hip dysplasia, thoracic deformities, genu valgum and metaphyseal dysplasia) and skin abnormalities (mainly hypopigmentation and palmoplantar cutis laxa) at 63% each, as well as microcephaly (42%), facial dysmorphia (37%), dental abnormalities (32%), and osteopenia/osteoporosis (32%). Pancreas lipomatosis was reported in five cases (26%), with four out of the five patients suffering from exocrine pancreatic dysfunction with preserved endocrine function. Intriguingly, while (high) myopia, astigmatism and other visual field defects were described in six patients (32%), rare features of retinal (rod-cone) dystrophy and other retinal abnormalities were also identified in six patients (32%), albeit one patient developed symptoms after HSCT (65) and another patient carried a homozygous variant in *PCARE*, known to cause an autosomal recessive form of retinitis pigmentosa (**Table 1**) (65). This suggests retinal abnormalities may be part of the developing phenotype. Due to the low number of reported patients, specific phenotypic features need to be defined and refined over time.

Sixteen Italian patients with Shwachman-Diamond syndrome and bi-allelic *SBDS* variants were screened for additional variants in *DNAJC21*, *EFL1*, and *SRP45*. One of the two germline-mutated *SBDS* patients with compound heterozygous *SBDS* variants and an additional heterozygous *DNAJC21* variant was reported to suffer from a more severe hematologic phenotype, in particular severe neutropenia (70). Both *DNAJC21* variants identified in this study (70), E276K and V342M, are reasonably rare in 0,32% and 0,0016% of the gnomAD population but ensemble *in-silico* predictions are contradicting or in favor of no significant impact on the protein structure.

## Digenic ADH5/ALDH2 deficiency causes BMF through defective formaldehyde detoxification

Formaldehyde is a ubiquitous endogenous and environmental metabolite, which has been classified as a group I human carcinogen by the International Agency for Research on Cancer as it may cause nasopharyngeal cancer, lung cancer and is associated with development of AML (71). Specifically, it was shown that formaldehyde exposure induces chromosomal aneuploidy, in particular aberrations of chromosomes 5, 7, and 8, which are frequently seen in AML (72, 73). It was also reported to induce hematopoietic toxicity to both mature and stem/progenitor cells in the bone marrow of mice exposed to formaldehyde by affecting myeloid progenitor growth and survival through oxidative damage apoptosis and dysregulation of colony stimulating factor receptors (74).

**Figure 4:**
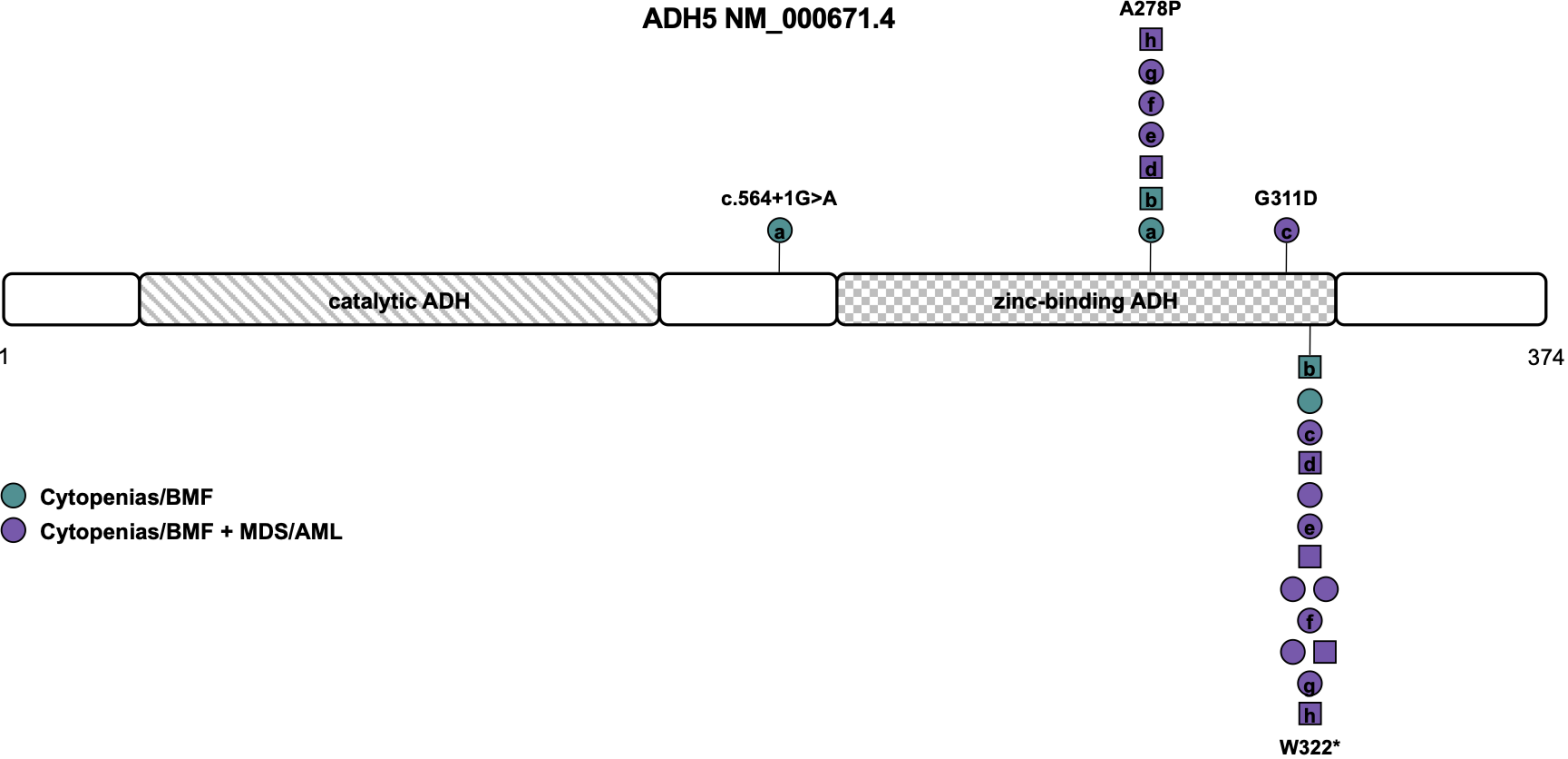
Schematic of the *ADH5* transcript (NM_000671.4) and its protein domains with the location of all reported germline variants. Circles represent females and squares represent males. Symbols on the same horizontal level are individuals from the same family. Letters within the symbols indicate individuals with compound heterozygous genotype, while symbols without letters indicate homozygosity for the variant. The color fill depicts different phenotypes. ADH, alcohol dehydrogenase; AML, acute myeloid leukemia; BMF, bone marrow failure; MDS, myelodysplastic syndrome.

ADH5, located in the cytoplasm, is the most widely expressed alcohol dehydrogenase and the main formaldehyde-detoxifying enzyme (75). It contains a catalytic and zinc-binding domain of the alcohol dehydrogenase (**Figure 4**). ALDH2, a mitochondrial enzyme oxidizing acetaldehyde to acetate, is important in ethanol metabolism, and deficiency of this enzyme is very common in humans, leading to facial flushing, nausea, headaches, cardiac palpitations, and overall discomfort in response to drinking alcohol (76). The ALDH2*2 allele, defined by the c.1510G>A (NM_000690.4) variant (rs671), encoding an E504K amino acid substitution, reduces enzyme activity to less than 50% in heterozygotes and less than 4% in homozygotes in a dominant-negative manner (76). It is common in the East Asian population with an allele frequency of about 25% in the gnomAD (https://gnomad.broadinstitute.org/) population. ALDH2, active mainly in detoxifying acetaldehyde, also takes part in formaldehyde detoxification (75).

Dingler et al. described the first seven children/young adults with homozygous or compound heterozygous *ADH5* variants and the heterozygous ALDH2*2 allele associated with BMF and predisposition to MDS/AML that is solely driven by formaldehyde accumulation (16). A subsequent study by Oka et al. reported seven individuals from five different families with BMF and development of MDS/AML at young age (77). Of those, four individuals were heterozygous and three homozygous for the ALDH2*2 allele. The three homozygotes were reported to harbor more severe phenotypes including neurological deterioration and early death (77). Because of its association with short stature and intellectual disability, it was subsequently also called AMeD syndrome (for Anemia, Mental retardation and Dwarfism) (10). One male patient with a history of anemia since the age of 8 years who developed MDS at 18 years old was diagnosed with ADH5/ALDH2 deficiency and sensorineural hearing loss based on concurrent compound heterozygous variants in *ADGRV1* (78).

Of the 15 individuals from 13 families described so far, 8 were compound heterozygous and seven homozygous for variants in *ADH5.* Consanguinity or occurrence of *de novo* variants was not reported (**Table 1**). Causative variants include LOF variants c.564+1G>A (NM_000671.4), resulting in retention of intron 5 (L188fs), and the recurrent variant W322*, that has been described in all but one individual (**Table S1**). Missense variants were located within the zinc-binding domain of ADH5, namely the G311D variant and the recurrent A278P variant found in seven patients (**Tables 1**, **S1** and **Figure 4**) (16, 77, 78). Penetrance is complete with all patients diagnosed with either BMF or early-onset MDS/AML and nine out of twelve patients (75%) undergoing HSCT (**Table 1**). Based on the published data, MDS/AML was diagnosed in 12 out of 15 patients (80%) at an average age of 7 years (range 0 to 18 years (n=9), **Table 1**, **Figure 4**). In eight out of eleven patients (73%) with reported cytogenetic information, a gain of the long arm of chromosome 1, frequently seen in Fanconi anemia (79, 80) was described. Other recurrent cytogenetic alterations included monosomy 7, trisomy 8 and 21p alterations (**Table 1**) (16, 77, 78). All patients with reported additional phenotypical data were short of stature and displayed mild to moderate intellectual disability. Microcephaly was described in 67% and abnormal skin pigmentation was found in 58% of patients (**Table 1**). Although the phenotype mimics Fanconi anemia, radial ray defects have not been detected so far and chromosomal breakage tests are negative. Other features such as retinal degeneration, facial dysmorphia, skeletal, and endocrine abnormalities have only been described in single individuals so that the clinical association remains unclear to date (16, 77, 78).

Using a CRISPR-Cas9 functional screen, *ADH5* was (together with *ESD* and the *FANC* family genes) described as a top candidate gene dramatically increasing cellular formaldehyde sensitivity when disrupted (81). Concordantly, *Adh*5^-/-^ *Aldh2* ^-/-^ double-deficient mice recapitulated some of the hematopoietic phenotypes seen in these patients such as reduced proliferation of HSPCs and loss of differentiation (16, 77). Another group reported that *Adh5* ^-/-^ deficient mice with wildtype Aldh2 are born and develop normally, while double-deficient mice showed significantly lower body weight, which mimics the short stature seen in humans (82).

Formaldehyde also triggers a cellular redox imbalance that can lead to reactive oxygen species accumulation and cytotoxicity, which may cause BMF development even in the presence of functional DNA repair mechanisms by overwhelming the DNA-repair capacity in HSPCs (83). Using patient-derived lymphoblasts, fibroblasts, induced pluripotent stem cells (iPSCs), and CRISPR/Cas9-engineered cell lines, Mu et al. were able to demonstrate that patient-derived iPSCs were sensitive to exogenous treatment with formaldehyde, which induced drastically defective cell expansion when stimulated into hematopoietic differentiation and increased levels of DNA damage. This phenotype was attenuated upon expression of *ADH5* and less so by addition of an ALDH2 activator (84). Therapies aiming to lower endogenous formaldehyde could be a promising treatment strategy for ADH5/ALDH2 deficiency. C1, a new small molecule acting as agonist of ALDH2, was well tolerated and able to partially reverse the HSPC expansion/differentiation defect in iPSCs *in vitro* (84). The combination of a formaldehyde scavenger such as metformin and glutathione precursors (for instance N-acetyl-L-cysteine) (83) may also benefit patients with Fanconi anemia.

In whose transcriptional reprogramming during differentiation of HSPCs may lead to acute accumulation of endogenous DNA damage, most likely arising from formaldehyde, an obligate by-product during transcriptional regulation (85). Further studies are needed to determine if aldehydes are the major cause of pathology in Fanconi anemia patients, who have functional ALDH2 and ADH5 to mediate aldehyde metabolism.

## Discussion

There may be a confounding bias for all described syndromes by the short period of clinical observations since these syndromes have been discovered. The likelihood of developing hematologic malignancies and the penetrance of these diseases may be estimated as too low since many patients are still children or young adults and others already underwent HSCT to treat severe early-onset BMF.

Suspicion of an inherited BMF syndrome should arise when patients are diagnosed with BMF in infancy/early-childhood (7) and/or MDS at young age (below 40 years old) (29, 30). A positive family history and other organ manifestations also point towards an inherited rather than acquired BMF syndrome (2, 7, 17). Germline BMF panel-based next-generation sequencing (NGS) is a reasonable first-tier option (86). WES or whole-genome sequencing (WGS) should be considered when suspicion of an inherited BMF syndrome is high and initial panel-based results are negative. WES covers all coding genes, however, if genes have not been described as candidate genes at the time of the analysis, the genetic variant causing the phenotype may be missed. Regular re-analysis of WES, as was done in one patient with BMF and bi-allelic *DNAJC21* variants (66) should be included (87). Even if this initially may only increase the number of variants of unknown significance (88), these could be upgraded over time when new information such as observation in multiple probands, segregation with disease, or functional impact of the variant becomes available. Intragenic and whole-gene CNVs were described in *DNAJC21* (65), *ERCC6L2* (24), and *MECOM* (46, 48–50), so that high-density microarrays or bioinformatic analysis of panel-based NGS/WES data have to be incorporated (89). Non-canonical, deeply intronic or exonic synonymous splice variants may require additional RNA sequencing to unravel the effects on splicing and prove pathogenicity of these unusual but not infrequent variants (89–91).

Crucially, unrecognized inherited BMF syndromes may lead to use of related donors carrying the same variants (29), as well as excessive death upon HSCT, which can be reduced using tailored conditioning regimens (92). Adapted non-myeloablative conditioning protocols have been used successfully as conditioning regimens in telomere biology disorders and Fanconi anemia (93, 94). A small case series of six patients with germline *MECOM* variants reported that reduced-intensity conditioning was an effective treatment and reduced toxicity-related morbidities (95). However, comprehensive HSCT data from patients with germline *ERCC6L2, MECOM, DNAJC21*, and *ADH5/ALDH2* variants are lacking to date, including donor choice, conditioning regimens and non-relapse morbidity and mortality.

A clear genotype-phenotype correlation has so far only been established for *MECOM* variants, where the co-presentation of RUS and hematologic disease appears to be caused by variants spanning zinc fingers 8 and 9. Although there is indication that homozygosity for the ALDH2*2 allele may lead to a more severe (neurological) phenotype in patients with ADH5/ALDH2 deficiency (77), the number of individuals is too low to draw comprehensive conclusions at this time. The discovery of additional mechanisms of disease and amino acid hotspots may help predict the individual, variant-based risk of hematologic malignancies, severe BMF and other phenotypic features.

Some data suggest clonal genetic markers of disease progression such as (multi-hit) *TP53* variants in patients with *ERCC6L2* germline variants may be indicative of disease progression (24, 28–30) and given the poor prognosis of MDS/acute erythroid leukemia in these patients, early HSCT should be performed. Similar data have been reported for *TP53* variants in other inherited BMF syndromes (96, 97), although a clear link to disease progression in patients with somatic *TP53* variants in the absence of other high-risk molecular or cytogenetic markers is unclear (98). Early pre-emptive HSCT comes also with the potential of HSCT-related mortality and morbidity so that more valid early markers of disease progression are needed (99).

In summary, this review provides new insights into four distinct and recently described BMF syndromes and will thereby improve the clinical management for these patients. New data will over time further refine these syndromes and add more pieces to the yet unsolved puzzle of inherited BMF syndromes.

## Author contributions

SF conceptualized and designed the study and wrote the manuscript.
